# Enhanced blue-light excited cyan-emitting persistent luminescence of BaLu_2_Al_2_Ga_2_SiO_12_:Ce^3+^, Bi^3+^ phosphors for AC-LEDs via defect modulation

**DOI:** 10.1038/s41377-022-00868-8

**Published:** 2022-06-17

**Authors:** Weihong Yuan, Ran Pang, Shangwei Wang, Tao Tan, Chengyu Li, Chaowei Wang, Hongjie Zhang

**Affiliations:** 1 State Key Laboratory of Rare Earth Resource Utilization, Changchun Institute of Applied Chemistry, Chinese Academy of Sciences, Changchun, 130022 China; 2 University of Science and Technology of China, Hefei, 230026 China; 3 Zhongke Rare Earth (Guangzhou) Co., Ltd, Guangzhou, 510700 China

**Keywords:** Inorganic LEDs, Optical materials and structures

## Abstract

Alternating current light-emitting diodes (AC-LEDs) have received significant attention from both academia and industry due to their remarkable benefits of more compact volume, cheaper manufacturing cost, greater energy usage efficiency, and longer service life. One of the most significant challenges for AC-LEDs is the flicker effect, which is mainly caused by the unavoidable 5–20 ms dimming time. Aiming to reduce the flicker effect, we designed a series of excellent blue-light excited cyan-emitting persistent luminescence (PersL) phosphors BaLu_2_Al_2_Ga_2_SiO_12_:Ce^3+^, Bi^3+^ via defect engineering of co-doping Bi^3+^. Interestingly, we found that co-doping Bi^3+^ not only effectively enhanced the PersL intensity, but also regulated the PersL lifetime of this phosphors. As the Bi^3+^ co-doping concentration increases to 0.01, the τ_80_ value (the time when the PersL intensity decreases to 80% of the initial intensity) increases from 0.24 to 19.61 ms, which proves to be effective in compensating the flicker effect of AC-LEDs. A new method of generating white light emission during the dimming time through adding the blue-light excited cyan PersL phosphor to the original orange-red PersL phosphor was proposed and an AC-LED lamp with a decreased percent flicker of 48.15% was fabricated, which is significantly better than the other currently reported AC-LED devices based on PersL phosphors. These results demonstrate that BaLu_2_Al_2_Ga_2_SiO_12_:Ce^3+^, Bi^3+^ might be an attractive material for low-flicker AC-LEDs.

## Abstract (web summary)

Efficient blue-light excited cyan-emitting persistent luminescence phosphor BaLu_2_Al_2_Ga_2_SiO_12_:Ce^3+^, Bi^3+^ is designed and synthesized to effectively compensate for the flicker effect of AC LEDs.

## Graphical abstract

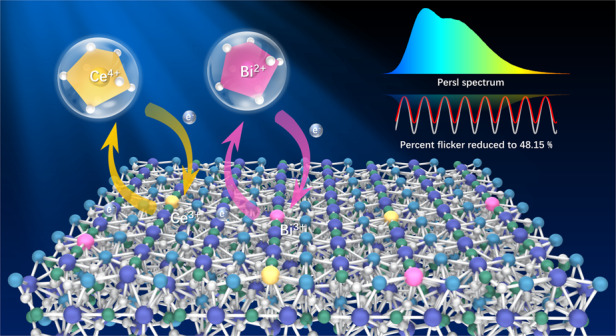

## Introduction

After decades of development, white light-emitting diodes (WLEDs) that can directly convert electrical energy into visible light outperform traditional incandescent and fluorescent lamps in the field of lifetime, luminous efficacy, and energy usage^1,2^. Nowadays, the municipal electric power is supplied in form of alternating current (AC), while most light-emitting diode (LED) semiconductor chips are powered by direct current (DC)^3^. Based on this situation, the compact AC-DC converter is an important electronic component that converts AC from city power to DC, which can be applied into WLEDs to ensure the operation of DC-LEDs^4^. However, the conversion from AC to DC consumes nearly 30% of the input electric power^5,6^. Furthermore, the AC-DC converter raises the price and expands the size, complicating the product appearance. The massive heat generated during the conversion process also causes degradation of the embedded phosphor, which severely shortens the service lifetime of WLEDs^7,8^. Thus, in view of these shortcomings of DC-LEDs, AC-LEDs have received widespread attention in both academic research and industrial applications owing to their remarkable benefits of more compact volume, cheaper manufacturing cost, greater energy usage efficiency, and longer service life^9,10^. Despite so many potential advantages, a pressing issue limiting the commercial application of AC-LEDs is the flicker effect, which is mainly caused by the unavoidable 5–20 ms dimming time in each AC cycle. This effect may also induce headaches, eyestrain, and other symptoms in certain people^11,12^. Consequently, reducing the flicker effect has become the key issue for the further applications of AC-LED lighting systems. As we all know, the fabrication of WLEDs generally involves combining LED chips with phosphors, which provides a method to overcome this problem. PersL phosphors can absorb and store excitation energy and then continuously emit luminescence for an appropriate duration from few seconds to several hours after ceasing the irradiation^13,14^. Considering that PersL phosphors can maintain luminescence for a certain period of time, they have excellent application prospects to minimize the flicker effect of AC-LEDs by employing the PersL to compensate the dark duration^15,16^. Charging and detrapping are the two fundamental processes involved in the PersL phenomenon^17,18^. To satisfy the criteria of AC-LED applications, both processes must be taken into account. First, the electron charging process refers to that under high-energy radiation, electrons can be excited from the ground-state energy level of the emission center to the conduction band (CB) and captured by traps. Accordingly, PersL phosphors should have intensed absorption of blue-light to match commercial InGaN chips^19–21^. Besides, the electron detrapping process is related to the recombination of the trapped electrons with the holes at emission centers, and thus, the depth and concentration of traps should be suitable to ensure strong PersL intensity within a decay time of 5–20 ms.

The strategy of using PersL phosphor to reduce the flicker effect in AC-LEDs was first proposed by our group^22^. Thereafter, a serial of yellow PersL phosphors excited by blue-light such as Lu_2_CaMg_2_(Ge_*x*_, Si_1-*x*_)_3_O_12_:Ce^3+^, Gd_3_Al_2_Ga_3_O_12_:Ce^3+^, and Mg_3_Y_2_(Ge_*x*_, Si_1-*x*_)_3_O_12_:Ce^3+^ have been reported and are expected to be applied in AC-LEDs, and they can reduce the percent flicker to 64.1%, 69%, and 71.7%, respectively^19–21^. Among them, Lu_2_CaMg_2_(Ge_*x*_, Si_1-*x*_)_3_O_12_:Ce^3+^ and Mg_3_Y_2_(Ge_*x*_, Si_1-*x*_)_3_O_12_:Ce^3+^ are focused on their millisecond PersL performance and the results preliminarily demonstrate that millisecond PersL has the potential to compensate for the flicker. Due to the absence of blue-light component during the dimming time, the AC-LEDs fabricated using blue chips and yellow PersL phosphors usually exhibit a severe shortcoming that only yellow light is compensated during the dimming time. White light can be obtained from the AC-LEDs while the voltage is above than the turn-on voltage (*V*_*f*_), but when the voltage falls below *V*_*f*_, only yellow light is left. More concretely, the white luminescence of AC-LEDs is momentary rather than constant. To overcome this situation, a new solution of adding blue-light excited blue PersL phosphor to the original yellow PersL phosphor to generate white light emission during the dimming time is proposed. However, contrary to the steady advancement of research focusing on PersL phosphors in the yellow region, the research on blue PersL phosphors that can be used in AC-LEDs is still scarce. Therefore, it is critical to develop and synthesize outstanding blue PersL phosphors with high intensity, appropriate duration, and effective excitation by blue-light.

Herein, we synthesized a series of excellent blue-light excited cyan PersL phosphor by co-doping Bi^3+^ into Ce^3+^ activated BaLu_2_Al_2_Ga_2_SiO_12_. The phosphors exhibit cyan emission under 425 nm excitation, and intense cyan PersL can be observed after ceasing the excitation. Meanwhile, we found that co-doping Bi^3+^ not only effectively enhanced the PersL intensity, but also regulated the PersL lifetime of this phosphors. As the Bi^3+^ co-doping concentration increases to 0.01, the value of τ_80_ increases from 0.24 to 19.61 ms. Aiming to minimize the flicker effect and compensate for the absence of blue-light component during the dimming time in each AC cycle, an AC-LED lamp was fabricated using commercial orange-red PersL phosphor Sr_0.75_Ca_0.25_S:Eu^2+^ (SCS:Eu^2+^) and as-prepared phosphor BaLu_2_Al_2_Ga_2_SiO_12_:0.05Ce^3+^, 0.01Bi^3+^ on a commercial blue-emitting chip. The device exhibits intense white light when the current is set to 20 mA and shows bright white PersL emission during the dimming time with a decreased percent flicker of 48.15%, which is significantly better than the other currently reported AC-LED devices based on PersL phosphors. These results demonstrate that BaLu_2_Al_2_Ga_2_SiO_12_:Ce^3+^, Bi^3+^ might be a promosing blue-light excited cyan PersL phosphor for low-flicker AC-LEDs.

## Results

### Crystal structure and micromorphology

**Fig. 1:**
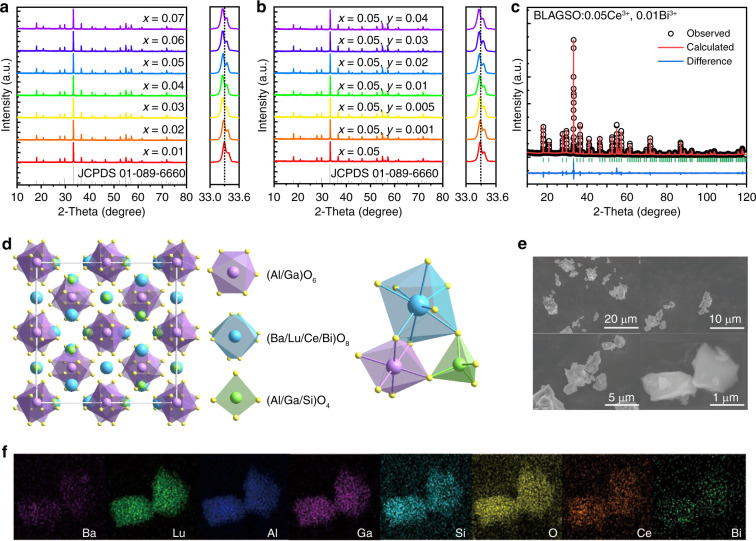
Crystal structure and micromorphology. **a** XRD patterns of BLAGSO:*x*Ce^3+^. **b** XRD patterns of BLAGSO:0.05Ce^3+^, *y*Bi^3+^. **c** XRD Rietveld refinement for BLAGSO:0.05Ce^3+^, 0.01Bi^3+^. **d** Crystal structure. **e** SEM of BLAGSO:0.05Ce^3+^, 0.01Bi^3+^. **f** Elemental mapping of BLAGSO:0.05Ce^3+^, 0.01Bi^3+^

X-ray diffractometer (XRD) patterns of BaLu_2-*x*_Al_2_Ga_2_SiO_12_:*x*Ce^3+^ (BLAGSO:*x*Ce^3+^) and BaLu_1.95-*y*_Al_2_Ga_2_SiO_12_:0.05Ce^3+^, *y*Bi^3+^ (BLAGSO:0.05Ce^3+^, *y*Bi^3+^) are exhibited in Fig. 1a, b, respectively. Clearly, there is a good correlation between the diffraction peaks and the Y_3_Ga_2_Al_3_O_12_ standard pattern (JCPDS 01-089-6660), confirming that pure phase was synthesized in all samples. With the augment of Ce^3+^ and Bi^3+^ concentrations, the magnified diffraction peak gradually shifts toward lower 2*θ* value side, which corresponds to the lattice expansion caused by Lu^3+^ (CN = 8, *r* = 0.977 Å) replaced by Ce^3+^ (CN = 8, *r* = 1.143 Å) and Bi^3+^ (CN = 8, *r* = 1.17 Å)^23^. To further corroborate the phase purity, the Rietveld refinements for BLAGSO, BLAGSO:0.05Ce^3+^, and BLAGSO:0.05Ce^3+^, 0.01Bi^3+^ were carried out and the results are shown in Figs. S1 and 1c. The obtained *R*_*wp*_ and *R*_*p*_ converge to 6.03% and 4.50% for BLAGSO, 6.06% and 4.52% for BLAGSO:0.05Ce^3+^, 6.21% and 4.60% for BLAGSO:0.05Ce^3+^, 0.01Bi^3+^, respectively, which implies that the refined results are highly credible. Tables S1–4 list the other detailed Rietveld refinement results. The as-prepared BLAGSO:0.05Ce^3+^, 0.01Bi^3+^ phosphor crystallizes in a cubic structure with space group Ia-${{{\bar{\mathbf 3}}}}$d (230). The refined parameters for the lattice are *a* = *b* = *c* = 12.0648 Å, *V* = 1756.137 Å^3^. In comparison to BLAGSO (*a* = *b* = *c* = 12.0483 Å, *V* = 1748.952 Å^3^) and BLAGSO:0.05Ce^3+^ (*a* = *b* = *c* = 12.0620 Å, *V* = 1754.924 Å^3^), the increased lattice parameters of BLAGSO:0.05Ce^3+^, 0.01Bi^3+^ also reveal that Bi^3+^ and Ce^3+^ successfully occupied the Lu^3+^ site. The crystal structure is shown in Fig. 1d, which demonstrates that the highly symmetrical crystal structure of BLAGSO:0.05Ce^3+^, 0.01Bi^3+^ contains three kinds of polyhedrons: [(Al/Ga/Si)O_4_] tetrahedron, [(Al/Ga)O_6_] octahedron, and [(Ba/Lu/Ce/Bi)O_8_] dodecahedron. [(Al/Ga/Si)O_4_] tetrahedrons and [(Al/Ga)O_6_] octahedrons are linked by sharing O^2−^ points. They also share edges or corners with [(Ba/Lu/Ce/Bi)O_8_] dodecahedrons to create a complex three-dimensional network.

Scanning electron microscope (SEM) images of BLAGSO:0.05Ce^3+^, 0.01Bi^3+^ are depicted in Fig. 1e, which exhibit irregular grain morphology and particle sizes. Figure 1f shows the elemental mapping images, which certify that all elements in the sample are homogeneously dispersed without any aggregation throughout the whole particle.

### Electronic structure

**Fig. 2:**
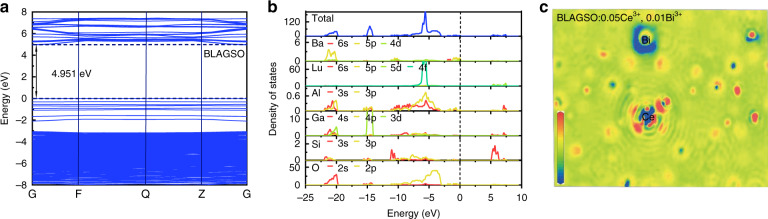
Electronic structure. **a** Band structure of BLAGSO. **b** DOS of BLAGSO. **c** Charge density difference of Ce and Bi in BLAGSO:0.05Ce^3+^, 0.01Bi^3+^

Figure 2 exhibits the calculated results of BLAGSO:0.05Ce^3+^ when considering the site-preference strategy for Bi^3+^. The low doping concentration of Bi^3+^ in the host makes it challenging to determine site-preference in the experiment. First-principles calculations have been demonstrated to be reliable in determining the most likely location of Bi^3+^ substitution sites based on relative formation energies. After structural optimization, according to Eq. (1), Bi^3+^ is set to occupy the Lu^3+^ site with the lowest formation energy (−2.3143 eV) instead of the Ba^3+^ site with a higher formation energy (−1.2755 eV)^24^. This result indicates that Bi^3+^ preferentially occupies the Lu^3+^ site.

1 $$\begin{array}{ll}E_f = E_{{\rm{total}}} - n_{{\rm{Ba}}}\mu _{{\rm{Ba}}} - n_{{\rm{Lu}}}\mu _{{\rm{Lu}}} - n_{{\rm{Al}}}\mu _{{\rm{Al}}} - n_{{\rm{Ga}}}\mu _{{\rm{Ga}}}\\\qquad - n_{{\rm{Si}}}\mu _{{\rm{Si}}} - n_{\rm{O}}\mu _{\rm{O}} - n_{{\rm{Ce}}}\mu _{{\rm{Ce}}} - n_{{\rm{Bi}}}\mu _{{\rm{Bi}}}\end{array}$$

where *E*_*f*_ is the formation energy, *E*_total_ reflects the total density functional theory (DFT) energy, *μ* refers to the atomic chemical potentials, and *n* means the atomic quantities.

The band structure of the host BLAGSO calculated using the generalized gradient approximation (GGA)-Perdew-Burke-Ernzerhof (PBE) formula is shown in Fig. 2a. Obviously, BLAGSO possesses a direct bandgap (*E*_*g*_) about 4.951 eV, since the maximum of valence band and the minimum of CB are placed at the G point. The discrepancy between experimental and calculated values is mainly due to the inherent shortcomings of the DFT-PBE methods. Partial density of state (DOS) can be used to further analyze the composition of the calculated band structure, as presented in Fig. 2b. The low-energy area around the Fermi level is mainly contributed by Ba-5p, Lu-4f, Ga-3d, O-2s2p states, while Lu-5d, Ga-4s, Si-3s3p states mainly dominate the CB. According to the DOS analysis, O-2p states play a critical part in the area surrounding the Fermi level because of the strong electronegativity of the O ion, and the Lu–O bond is formed by hybridization of O-2p with Lu-4f, which determines the optical properties of BLAGSO. Charge sharing and distribution can be predicted using charge density difference and bader charge. The charge density difference provides a visual picture of the change in charge distribution involving the introduction of Ce^3+^ and Bi^3+^ into BLAGSO (Fig. 2c). Here, the red color (positive) around the Ce atom indicates that Ce^3+^ loses electrons, while the blue color (negative) around the Bi atom indicates that Bi^3+^ gains electrons. Moreover, the bader charge of Bi is −2.03 eV in BLAGSO:0.05Ce^3+^, 0.01Bi^3+^, which is similar to the bader charge of −1.97 eV in BiO. Therefore, the above results confirm the presence of Bi^2+^, which also provides a basis for Bi^3+^ to act as electron trapping centers in BLAGSO:Ce^3+^, Bi^3+^.

### Luminescence properties

**Fig. 3:**
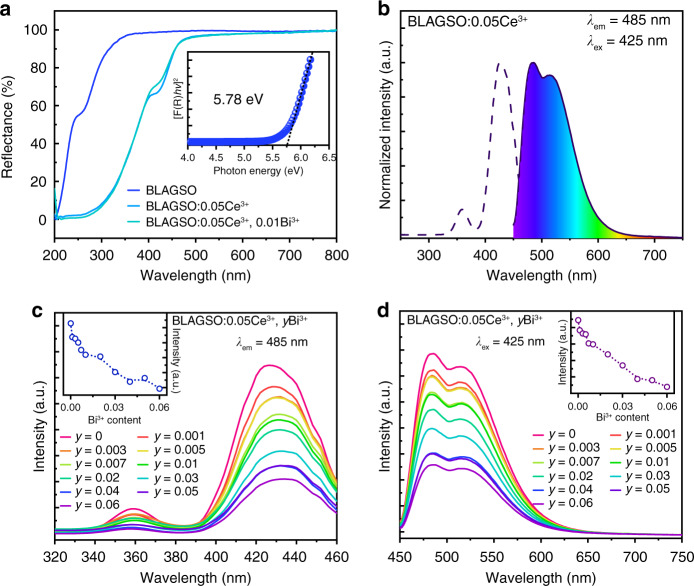
Photoluminescence properties. **a** DR spectra of BLAGSO, BLAGSO:0.05Ce^3+^, and BLAGSO:0.05Ce^3+^, 0.01Bi^3+^. Inset: the experimental *E*_*g*_ of BLAGSO. **b** PLE and PL spectra of BLAGSO:0.05Ce^3+^. **c** PLE spectra of BLAGSO:0.05Ce^3+^, *y*Bi^3+^. Inset: the PLE intensity of BLAGSO:0.05Ce^3+^, *y*Bi^3+^. **d** PL spectra of BLAGSO:0.05Ce^3+^, *y*Bi^3+^. Inset: the PL intensity of BLAGSO:0.05Ce^3+^, *y*Bi^3+^

The diffuse reflectance (DR) spectra of BLAGSO, BLAGSO:0.05Ce^3+^, and BLAGSO:0.05Ce^3+^, 0.01Bi^3+^ are presented in Fig. 3a. The undoped BLAGSO sample exhibits strong absorption located at 200–350 nm and high reflection between 350 and 800 nm. In the case of Ce^3+^ doped or Ce^3+^/Bi^3+^ co-doped BLAGSO, strong absorption bands appear at 200–500 nm, which are ascribed to Ce^3+^: 4f → 5d transition. The host experimental *E*_*g*_ was calculated based on the following equations^25^:

2 $$\left[ {F\left( R \right)hv} \right]^2 = C\left( {hv - E_g} \right)$$

3 $$\left[ {F\left( R \right)} \right] = \left( {1 - R} \right)^2/2R$$

where *hν* means the photon energy, *R* denotes reflectance coefficient, *C* is an absorption constant, and *E*_*g*_ represents the experimental bandgap. As presented in the inset of Fig. 3a, the experimental *E*_*g*_ is 5.78 eV.

Figure 3b shows the photoluminescence excitation (PLE) and photoluminescence emission (PL) spectra of BLAGSO:0.05Ce^3+^. The PLE spectrum consists of an intense excitation band between 390 and 470 nm peaking around 425 nm and a weak excitation band between 340 and 380 nm peaking at 360 nm, which originate from Ce^3+^: 4f → 5d_1_ and 4f → 5d_2_ transitions. BLAGSO:0.05Ce^3+^ exhibits a luminescence band within the 450–650 nm spectral region centered at 485 nm under 425 nm excitation, belonging to the Ce^3+^: 5d → 4f transition. As we all know, host lattice strongly influences Ce^3+^ emission. According to recent reports, replacing Lu^3+^-Al^3+^ with Ba^2+^-Si^4+^ in Lu_3_Al_5_O_12_:Ce^3+^ has the effect of reducing Stokes shift and causing a blue-shift in PL spectrum^26^. In addition, a reduction in crystal field splitting takes place due to the increased diameter of the ions substituted at the octahedral sites. Partial substitution of Al^3+^ with Ga^3+^ also causes a blue-shift in PLE and PL spectra. Because of the extremely symmetrical crystal structure of BLAGSO, the Stokes shift of Ce^3+^ emission is relatively small (2910 cm^−1^, 0.36 eV), making the realization of blue-light stimulated cyan-emitting PersL phosphor possible. Furthermore, the emission peak can be deconvoluted to two Gaussian peaks at 20,872 and 19,257 cm^−1^ with an energy difference of 1615 cm^−1^, as shown in Fig. S2, corresponding to the theoretical energy separation between the ^2^F_5/2_ and ^2^F_7/2_ levels of Ce^3+^^27,28^. Figure 3c, d depicts the PLE and PL spectra of BLAGSO:0.05Ce^3+^, *y*Bi^3+^. As the co-doping content of Bi^3+^ increases, the PL intensity of Ce^3+^ gradually declines under 425 nm excitation. It may be closely connected with the trapping of the excited 5d electrons of Ce^3+^. The trapped electrons cannot escape immediately resulting in the reduction of emission intensity. The internal quantum efficiencies (IQEs) of BLAGSO:0.05Ce^3+^ and BLAGSO:0.05Ce^3+^, 0.01Bi^3+^ are 92.2% and 48.6%, respectively (Fig. S3). Meanwhile the PLE and PL spectra of BLAGSO:0.05Ce^3+^, *y*Bi^3+^ have same shapes as those of BLAGSO:0.05Ce^3+^, indicating that the Ce^3+^ is the emission center in both Bi^3+^ doped and undoped samples, and there is no energy transfer between Ce^3+^ and Bi^3+^, which can be further demonstrated by the PLE and PL spectra of BLAGSO:0.01Bi^3+^ (Fig. S4).

**Fig. 4:**
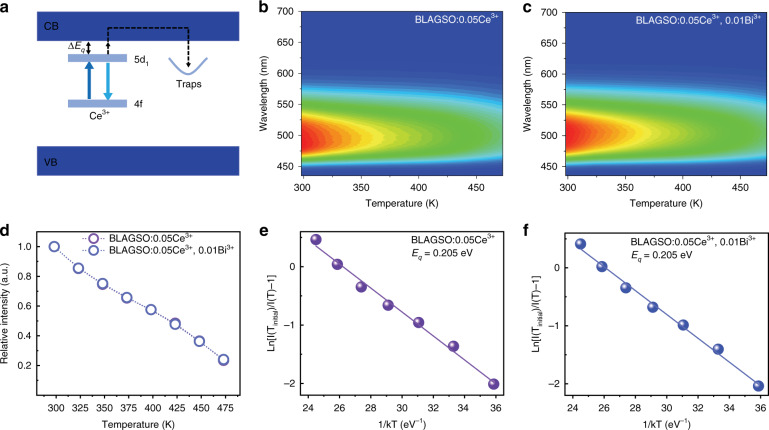
Temperature-dependent photoluminescence properties. **a** Thermal ionization process in BLAGSO:Ce^3+^ and BLAGSO:Ce^3+^, Bi^3+^. **b**, **c** The corresponding temperature-dependent PL spectra of BLAGSO:0.05Ce^3+^ and BLAGSO:0.05Ce^3+^, 0.01Bi^3+^. **d** Temperature-dependent PL intensity ratios *I*(*T*)/*I*(*T*_initial_) of BLAGSO:0.05Ce^3+^ and BLAGSO:0.05Ce^3+^, 0.01Bi^3+^. **e**, **f** The relation between Ln[*I*(*T*_initial_)/*I*(*T*) – 1] and 1/*kT* for BLAGSO:0.05Ce^3+^ and BLAGSO:0.05Ce^3+^, 0.01Bi^3+^

The d → f transition of Ce^3+^ is often accompanied by some non-radiative processes, such as thermal ionization and non-radiative crossover relaxation. Since the thermal ionization process is usually related to the charging process of PersL, the energy gap between the bottom of CB and the lowest Ce^3+^: 5d_1_ level is crucial for the PersL generation. It is actually the case that many Eu^2+^-doped phosphors exhibit thermal quenching caused by thermal ionization rather than crossover mechanism^29^. This quenching mechanism also applies to Ce^3+^, as illustrated in Fig. 4a^30^. In the case of thermal ionization causing the quenching process, the energy gap between CB and 5d_1_ level may be determined by analyzing and studying the temperature dependence of non-radiative decay rates. Due to the relatively small Stokes shift of BLAGSO:Ce^3+^ and BLAGSO:Ce^3+^, Bi^3+^, we can assume that the non-radiative 5d → 4f crossover relaxation has less effect on the thermal quenching. Thus, this approach can be used to evaluate the role of thermal ionization process of Ce^3+^ excited 5d electrons into CB for BLAGSO:Ce^3+^ and BLAGSO:Ce^3+^, Bi^3+^ in the PersL charging process. Figures 4b, c and S5 exhibit the temperature-dependent PL spectra of BLAGSO:0.05Ce^3+^ and BLAGSO:0.05Ce^3+^, 0.01Bi^3+^, respectively. Figure 4d shows the corresponding normalized PL spectra. One can see that the quenching process is strongly influenced by temperature and the temperature-dependence of the non-radiative decay rate is approximately the same for both samples. The energy barrier can be accurately modeled using the single barrier quenching function^31,32^:

4 $$I\left( T \right) = \frac{{I\left( {T_{ {\rm{initial}}}} \right)}}{{1 + \frac{{{{\Gamma }}_0}}{{{{\Gamma }}_v}}\exp \left( { - E_q/kT} \right)}}$$

where *I*(*T*_initial_) donates the initial luminescence intensity and *I*(*T*) is the intensity at temperature *T*, *Γ*_0_ refers to the attempt rate for the thermal ionization process and *Γ*_*v*_ represents the radiative decay rate, *k* is Boltzmann constant, *E*_*q*_ refers to the energy barrier for thermal ionization of Ce^3+^ emission. As presented in Fig. 4e, f, the *E*_*q*_ of BLAGSO:0.05Ce^3+^ and BLAGSO:0.05Ce^3+^, 0.01Bi^3+^ samples are estimated to be 0.205 eV. According to the results, co-doping Bi^3+^ has little effect on the distance between CB and Ce^3+^: 5d_1_ level, in other words, the possibility of Ce^3+^: excited 5d electrons entering the CB by thermal ionization is the same. Based on the above analysis, we find that the reason for the enhancement of PersL by co-doping Bi^3+^ may be closely related to the electron capture during charging process, and the explanation is mentioned in the next section. Obviously, the energy gap between CB and 5d_1_ level is about 0.205 eV, which is a suitable distance. First, due to its small energy gap, the excited 5d electrons are efficiently ionized into CB under blue-light excitation, as we expected, this phosphor can be charged by blue-light at RT, making it promising for AC-LED applications. Second, the energy gap is sufficient for recombination luminescence due to incomplete quenching at RT.

**Fig. 5:**
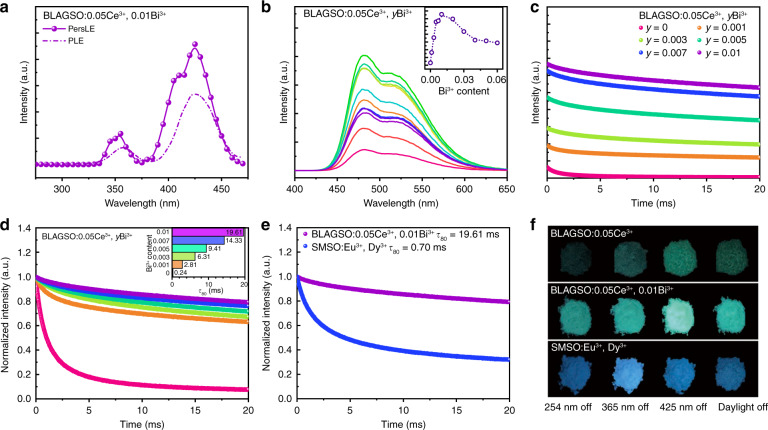
Persistent luminescence properties. **a** PersLE and PLE spectra of BLAGSO:0.05Ce^3+^, 0.01Bi^3+^. **b** PersL spectra of BLAGSO:0.05Ce^3+^, *y*Bi^3+^. Inset: the PersL intensity of BLAGSO:0.05Ce^3+^, *y*Bi^3+^. **c** PersL decay curves of BLAGSO:0.05Ce^3+^, *y*Bi^3+^. **d** Normalized PersL decay curves of BLAGSO:0.05Ce^3+^, *y*Bi^3+^. Inset: the values of τ_80_ for BLAGSO:0.05Ce^3+^, *y*Bi^3+^. **e** Normalized PersL decay curves of BLAGSO:0.05Ce^3+^, 0.01Bi^3+^ and SMSO:Eu^2+^, Dy^3+^. **f** PersL photographs of BLAGSO:0.05Ce^3+^, BLAGSO:0.05Ce^3+^, 0.01Bi^3+^, and SMSO:Eu^2+^, Dy^3+^ after excitation at 254, 365, 425 nm, and daylight, respectively

Garnet generated via high-temperature solid-state reaction generally exists plenty of localized defects, making garnet a potential candidate for PersL materials^20,33^. That is one reason why we chose the highly coordinated silicate-containing garnet BLAGSO:0.05Ce^3+^ for the study of AC-LEDs. To date, most blue PersL phosphors need to be effectively activated under ultraviolet or X-ray irradiation^34^. In this work, we find that BLAGSO:0.05Ce^3+^ exhibits weak PersL after ceasing the 425 nm blue-light irradiation, which concurs with the report of Lai et al, and remarkably, contrary to reducing the PL intensity, the Bi^3+^ co-doping significantly enhanced the PersL intensity of BLAGSO:0.05Ce^3+^^35^. The PersL excitation (PersLE) spectrum of BLAGSO:0.05Ce^3+^, 0.01Bi^3+^ shown in Fig. 5a, which was obtained by plotting PersL intensity versus excitation wavelength (Fig. S6a presents the representative PersL decay curves monitored at various excitation wavelengths), implies that BLAGSO:0.05Ce^3+^, 0.01Bi^3+^ can be successfully stimulated by blue-light. Meanwhile, the spectral patterns of PLE and PersLE are analogous, indicating that the charging process of PersL originates from Ce^3+^: 4f → 5d excitation, and the thermal ionization of Ce^3+^: 5d electrons into CB at RT is a crucial factor in the charging process as discussed above. The PersL spectra of BLAGSO:0.05Ce^3+^, *y*Bi^3+^ samples were recorded and exhibited in Fig. 5b to evaluate the effect of Bi^3+^ on the PersL performance. Obviously, the shapes of both PersL and PL spectra are found to be similar, demonstrating the origination of PersL is also from 5d → 4f transition of Ce^3+^. As Bi^3+^ co-doping concentration increases, the PersL intensity raises first until 0.01 and then declines. The rise in PersL intensity may be caused by the release of more captured electrons to the 5d_1_ level, while the quenching phenomenon related to the Bi^3+^ co-doping concentration is owing to the strengthen interaction of adjacent traps. This interaction can be interpreted by electron tunneling among traps at high Bi^3+^ concertrations^36^. Figure 5c exhibits the PersL decay curves of BLAGSO:0.05Ce^3+^, *y*Bi^3+^ within 20 ms after ceasing the 425 nm excitation, and their initial intensity corresponds to the PersL intensity. The normalized PersL decay curves are depicted in Fig. 5d, from which it can be seen that co-doping Bi^3+^ can regulate the PersL lifetime of this phosphors. The value of τ_80_ increases from 0.24 to 19.61 ms as the Bi^3+^ co-doping concentration increases to 0.01. Clearly, the τ_80_ value of BLAGSO:0.05Ce^3+^, 0.01Bi^3+^ is large enough to meet the requirement of AC-LEDs for dimming time^9^. When compared with the best known blue-emitting PersL phosphor Sr_2_MgSi_2_O_7_:Eu^2+^, Dy^3+^ (SMSO:Eu^2+^, Dy^3+^), BLAGSO:0.05Ce^3+^, 0.01Bi^3+^ exhibits stronger PersL intensity and superior PersL duration after 425 nm blue-light irradiation, and the τ_80_ value of BLAGSO:0.05Ce^3+^, 0.01Bi^3+^ is far larger than that of SMSO:Eu^2+^, Dy^3+^ (about 0.70 ms), as presented in Figs. 5e and S6b^37^. Figure 5f shows the PersL photographs of BLAGSO:0.05Ce^3+^, BLAGSO:0.05Ce^3+^, 0.01Bi^3+^, and SMSO:Eu^2+^, Dy^3+^ after excitation at 254, 365, 425 nm, and daylight, respectively. We can clearly see that the PersL intensity of BLAGSO:0.05Ce^3+^ is significantly enhanced by co-doping Bi^3+^, and the best excitation band for the PersL of SMSO:Eu^2+^, Dy^3+^ is located in the ultraviolet region, which makes it difficult to be applied in the field of AC-LEDs based on blue chips.

**Fig. 6:**
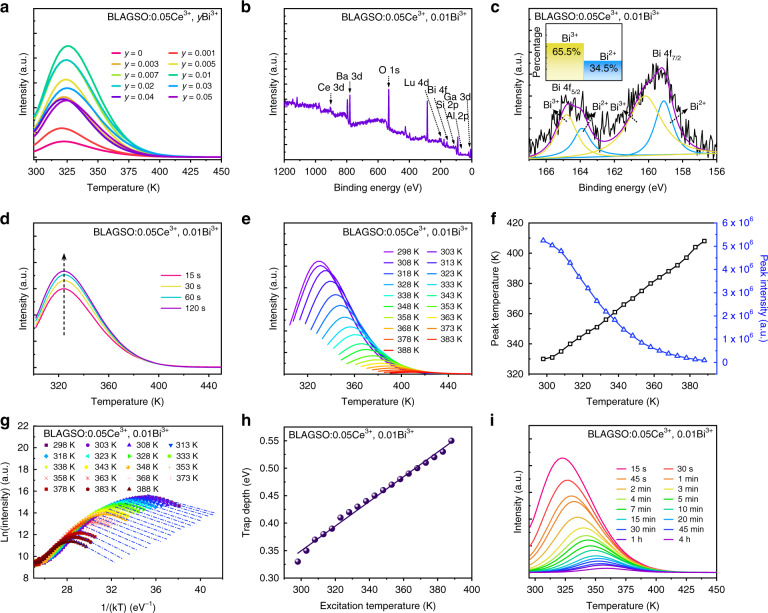
Thermoluminescence properties. **a** TL curves of BLAGSO:0.05Ce^3+^, *y*Bi^3+^ measured after 425 nm irradiation for 5 min. **b** XPS survey scan of BLAGSO:0.05Ce^3+^, 0.01Bi^3+^. **c** XPS analysis of Bi 4f. Inset: the concentration percentages of Bi^2+^ and Bi^3+^. **d** Excitation duration-dependent TL curves of BLAGSO:0.05Ce^3+^, 0.01Bi^3+^. **e** Excitation temperature-dependent TL curves of BLAGSO:0.05Ce^3+^, 0.01Bi^3+^. **f** Dependency on the *T*_exc_ for *I*_peak_ and *T*_peak_. **g** Initial rise analysis of BLAGSO:0.05Ce^3+^, 0.01Bi^3+^. **h** Relationship between calculated trap depth and *T*_exc_. **i** Decay duration-dependent TL curves of BLAGSO:0.05Ce^3+^, 0.01Bi^3+^

To better understand the mechanism of PersL in BLAGSO:Ce^3+^, Bi^3+^, the trap charging and detrapping processes were comprehensively investigated by a series of thermoluminescence (TL) measurements. The TL technique is the most effective method to study trap properties, such as trap distribution, depth, and density, as well as the interaction between electron trapping centers and emission centers in PersL phosphors. Figure 6a depicts the Bi^3+^ concentration-dependent TL spectra. The distribution of TL bands correlates with the trap depth, and the TL peaks mainly locate in the low-temperature region revealing the shallow depth of most traps. As can be seen, when the Bi^3+^ co-doping concentration is 0.01, the TL intensity reaches its maximum and subsequently drops as the concentration rises. Also noteworthy, the position and intensity of TL peak are strongly influenced by Bi^3+^ concentration, suggesting that the doping of Bi^3+^ promotes trap creation and affects trap concentration. According to Dorenbos et al., Bi^3+^ can act as both electron traps to form Bi^2+^ and hole traps to form Bi^4+^, and considering the previous discussion on the charge density difference, bader charge, and thermal ionization process of Ce^3+^, we tend to think that Bi^3+^ ions serve as electron trapping centers in BLAGSO:Ce^3+^, Bi^3+^ phosphors^38,39^. To further confirm this mechanism, the existence of Bi^2+^ was confirmed using X-ray photoelectron spectroscopy (XPS) investigation. Figure 6b illustrates the XPS survey scan of BLAGSO:0.05Ce^3+^, 0.01Bi^3+^, where all elements can be observed. The corresponding high-resolution Bi 4f XPS scan exhibits two binding energy peaks corresponding to the Bi 4f_5/2_ and 4f_7/2_, respectively. As shown in Fig. 6c, after deconvolution, the peaks at 160.2 eV, 164.8 eV are attributed to Bi^3+^, while the peaks at 159.1 eV, 163.9 eV are attributed to Bi^2+^, and the ratios of Bi^3+^ and Bi^2+^ components are about 65.5% and 34.5%, respectively^40–42^. Accordingly, we confirm the presence of Bi in the mixed oxidation state, and further verify the role of Bi^3+^ acting as the electron trapping centers in the BLAGSO:Ce^3+^, Bi^3+^ phosphors. Moreover, oxygen vacancie ${{{\mathrm{V}}}}_{{{{\ddot{\mathrm O}}}}}$ may be generated during the sintering process, which also contributes to the occurrence of PersL^43^.

**Fig. 7:**
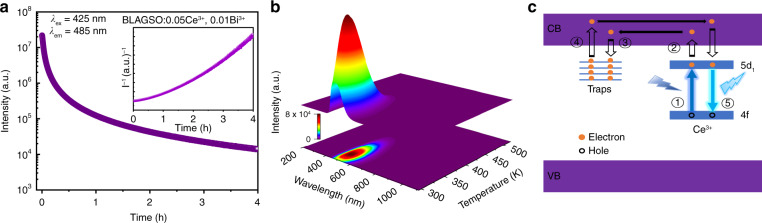
Persistent luminescence mechanism for BLAGSO:0.05Ce^3+^, 0.01Bi^3+^. **a** PersL decay curve. Inset: the function of *I*^−1^ versus *t*. **b** 3D-TL spectrum. **c** PersL mechanism

A sequence of irradiation duration-dependent TL tests was carried out on BLAGSO:0.05Ce^3+^, 0.01Bi^3+^ and the results are demonstrated in Fig. 6d. The TL intensity enhances as the excitation time increases since more electrons are trapped. The positions of all the TL peaks present no appreciable shift for different exposure time. This demonstrates that the electron escaping from traps conforms to first-order kinetic, which means that every electron released from the traps immediately recombines with the emission center to produce luminescence without being recaptured by the traps^44^. The first-order TL curves generally exhibit asymmetric glow peaks with negative slope, however, the curve profiles shown in Fig. 6d are approximately symmetric and relatively wide, suggesting the possibility of an overlap of various peaks with close trap depth distributions^45^. To further investigate the trap depth, we carried out a sequence of excitation temperature (*T*_exc_)-dependent TL analysis on BLAGSO:0.05Ce^3+^, 0.01Bi^3+^, as illustrated in Fig. 6e^46^. These analyses were conducted in accordance with the Eeckhout’s method. Each measurement involved heating the sample to a specified *T*_exc_, excited by 425 nm blue-light for 3 min, and stabilized for 3 min before starting measurements at 3 K s^−1^ rising rate from *T*_exc_. Stabilizing keeps the traps from being affected by the unstable electrons and the rapid decay process. Thus, the trap properties obtained by TL curves reveal the state of traps occupied by stable electrons under the different *T*_exc_. The plot of TL peak intensity (*I*_peak_) and TL peak temperature (*T*_peak_) versus *T*_exc_ is shown in Fig. 6f. The TL intensity reduces with rising *T*_exc_, along with the TL peak position shifts toward higher temperatures, mainly due to the thermal depletion of electrons. Since TL peak temperature reflects trap depth, TL peak shift in BLAGSO:0.05Ce^3+^, 0.01Bi^3+^ implies a continuous trap distribution. An initial rising method on the TL curves shown in Fig. 6g is used to further estimated trap depth. According to this method, the trapped electron concentrations are assumed to remain constant on low-temperature side, and thus, the TL intensity (*I*(*T*)) is unaffected by kinetics. This can be evaluated by the following formula^46,47^:

5 $$I\left( T \right) = C\exp \left( {\frac{{ - E}}{{kT}}} \right)$$

where *E* represents the trap depth, *k* denotes the Boltzmann constant, and *C* is the pre-exponential constant. *E* can be calculated from TL curves by plotting Ln(*I*) and 1/*T*. Clearly, as *T*_exc_ increases, the trap depths range between 0.30 and 0.56 eV. As we all know, trap depths of 0.2–0.5 eV are well suited for the generation of PersL. The trap depth in BLAGSO:0.05Ce^3+^, 0.01Bi^3+^ sample is highly compatible with ideal trap depth of PersL phosphors that can be applied in the field of AC-LEDs^29^. By further depicting the relationship between *T*_exc_ and estimated trap depth, the distribution of trap depth can be approximately determined, as shown in Fig. 6h. One can see that it follows a linear fit, indicating a constant detrapping rate, on the basis of which we can conclude that BLAGSO:0.05Ce^3+^, 0.01Bi^3+^ has a uniform trap depth distribution. Figure 6i exhibits the TL curves of BLAGSO:0.05Ce^3+^, 0.01Bi^3+^ measured at different decay durations after 425 nm blue-light excitation, and the change of TL curve profiles shows the electron detrapping process. As we can see, as decay time rises, TL peaks move to the higher temperature side. That means the electrons first escape from the shallow traps, followed by the slow release of electrons stored in deep traps. To clearly reveal the electron detrapping of PersL, Fig. 7a presents PersL decay curve of BLAGSO:0.05Ce^3+^, 0.01Bi^3+^. Apparently, in the beginning, the PersL intensity drops rapidly, then it slowly declines for several hours. Further, the inset of Fig. 7a exhibits the correlation between reciprocal PersL intensity (*I*^−1^) and time (*t*) converted from the PersL decay curve. The *I*^−1^–*t* curve deviates from a straight line, indicating that the electron detrapping process is due to thermal detrapping of electrons from traps into CB^48^. Figure 7b depicts the 3D-TL spectrum of BLAGSO:0.05Ce^3+^, 0.01Bi^3+^. From this spectrum, TL curves at various wavelengths and TL emission at various temperatures can be observed. Obviously, the TL emission of BLAGSO:0.05Ce^3+^, 0.01Bi^3+^ at different temperatures has a similar shape and position to the PersL emission.

On the basis of above results and analysis, we put forward a reasonable mechanism illustrated in Fig. 7c to explain the generation of the blue-light excited cyan PersL from BLAGSO:Ce^3+^, Bi^3+^ phosphor, which mainly consists of the following processes: ① excitation, ② electron ionization, ③ electron trapping, ④ electron detrapping, ⑤ electron recombination^17^. Electrons are stimulated from Ce^3+^: 4f ground state to 5d_1_ state when exposed to 425 nm blue-light during charging process. In view of the relatively small energy gap between CB and 5d_1_ level, plenty of excited electrons are easily thermally ionized from the 5d_1_ level to CB and subsequently captured by traps via CB. Bi^3+^ ions and ${{{\mathrm{V}}}}_{{{{\ddot{\mathrm O}}}}}$ can capture the escaping electrons, which serve as the electron trapping centers, and the holes are formed in the Ce^3+^: 4f ground state. The detrapping process involves releasing the trapped electrons to 5d_1_ level via CB after stopping the irradiation, and these electrons finally recombine with the holes in Ce^3+^, resulting in the occurrence of bright cyan PersL. The Bi^3+^ co-doping significantly enhanced both intensity and duration of PersL in Ce^3+^-doped BLAGSO phosphors, making it a potential candidate for AC-LED applications.

### Application of BLAGSO:0.05Ce^3+^, 0.01Bi^3+^ in AC-LEDs

**Fig. 8:**
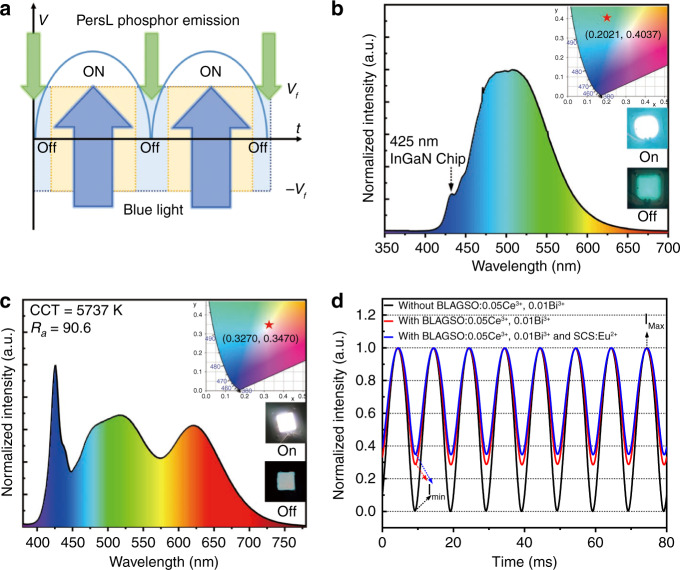
AC-LED applications for BLAGSO:Ce^3+^, Bi^3+^. **a** The scheme of AC flicker compensation using PersL phosphors. **b** EL spectrum of BLAGSO:0.05Ce^3+^, 0.01Bi^3+^. Inset: the CIE chromaticity coordinate and AC-LED device images on and off states. **c** EL spectrum of the white AC-LED fabricated by commercial orange-red PersL phosphor SCS:Eu^2+^, as-prepared phosphor BLAGSO:0.05Ce^3+^, 0.01Bi^3+^, and a 425 nm InGaN chip. Inset: the CIE chromaticity coordinate and AC-LED device images on and off states. **d** Luminescence intensity variation during AC cycle

For the purpose of evaluating the effectiveness of this PersL phosphor in reducing the flicker effect, we fabricated an AC-LED device by combining a 425 nm blue-emitting chip with BLAGSO:0.05Ce^3+^, 0.01Bi^3+^ based phosphor-in-silicone (PiS). Figure 8a exhibits the AC flicker compensation scheme using PersL phosphors. BLAGSO:0.05Ce^3+^, 0.01Bi^3+^ phosphor is excited to generate cyan-light at a voltage higher than *V*_*f*_, meanwhile, there are many electrons stored in the traps. When the excitation stops at a voltage lower than *V*_*f*_, the trapped electrons are released to produce PersL and the flicker effect of AC-LEDs is compensated. The corresponding electroluminescence (EL) spectrum is presented in Fig. 8b. Under a current of 20 mA, the device displays cyan emission with a CIE coordinate of (0.2021, 0.4037). Figure 8d shows the EL intensity variation at each AC cycle for the AC-LEDs with and without BLAGSO:0.05Ce^3+^, 0.01Bi^3+^. The flicker effect is usually expressed as the percent flicker (δ), which can be obtained according to the following formula^21^:

6 $$\delta = \frac{{I_{{\rm{max}}} - I_{{\rm{min}}}}}{{I_{{\rm{max}}} + I_{{\rm{min}}}}}$$

where *I*_max_ and *I*_min_ represent the maximum and minimum values of the luminous intensity, respectively. Obviously, the percent flicker of AC-LED is reduced from 100% to 55.04% owing to the compensation of PersL.

To further demonstrate that our solution of adding blue-light excited blue PersL phosphor to the original yellow PersL phosphor to generate white emission during the dimming time is reliable, we fabricated a white AC-LED device by using orange-red PersL phosphor SCS:Eu^2+^ and as-prepared phosphor BLAGSO:0.05Ce^3+^, 0.01Bi^3+^ on a 425 nm blue-emitting chip. As shown in Fig. 8c, the lamp displays bright white light with a CIE coordinate of (0.3270, 0.3470), the color render index (*R*_*a*_) of 90.6, and a correlated color temperature (CCT) of 5737 K under a current of 20 mA. It exhibits bright white PersL emission during the dimming time, and its percent flicker is reduced to 48.15% as presented in Fig. 8d. These results indicate that the cyan PersL phosphor BLAGSO:0.05Ce^3+^, 0.01Bi^3+^ has great potential for AC-LED applications.

## Discussion

In conclusion, we synthesized a sequence of excellent blue-light excited cyan-emitting PersL phosphors by introduced Bi^3+^ into BaLu_2_Al_2_Ga_2_SiO_12_:Ce^3+^. Benefiting from the highly symmetric crystal structure of BLAGSO, the phosphors show a relatively small Stokes shift of 0.36 eV. With a suitable energy gap between CB and Ce^3+^: 5d_1_ level, phosphor can be efficiently charged under 425 nm blue-light at RT. The co-doping of Bi^3+^ ions has little influence on the mechanisms of both PL and PersL, but can dramatically optimize the PersL lifetime. The τ_80_ value of BLAGSO:0.05Ce^3+^, 0.01Bi^3+^ is 19.61 ms, which proves to be effective in compensating the flicker effect of AC-LEDs. Based on the unique PersL properties of the phosphors, a new method of generating white light emission during the dimming time through adding blue-light excited blue PersL phosphor to the original yellow PersL phosphor was proposed, and an AC-LED lamp was fabricated using orange-red PersL phosphor SCS:Eu^2+^ and as-prepared phosphor BLAGSO:0.05Ce^3+^, 0.01Bi^3+^ on a 425 nm blue-emitting chip, the percent flicker of which was reduced from 100% to 48.15%. These results demonstrate that BLAGSO:Ce^3+^, Bi^3+^ might be a promising material for low-flicker AC-LEDs with the remarkable benefits of more compact volume, cheaper manufacturing cost, greater energy usage efficiency, and longer service life.

## Materials and methods

### Materials and preparation

A sequence of BLAGSO:*x*Ce^3+^ and BLAGSO:0.05Ce^3+^, *y*Bi^3+^ were produced via traditional high-temperature solid-state reaction. The starting ingredients included Bi_2_O_3_ (99.999%), Ce_2_(CO_3_)_3_ (99.999%), SiO_2_ (A.R.), Ga_2_O_3_ (99.99%), Al_2_O_3_ (99.99%), Lu_2_O_3_ (99.99%), BaF_2_ (A.R.), and BaCO_3_ (A.R.). Following the weighing of the starting ingredients based on the stoichiometric proportions, we mixed and ground the ingredients for 20 min in agate mortars. The resulting mixture was transferred to alumina crucibles and heated at 1400 °C for 5 h under N_2_ environment. After being cooled to RT, the synthesized sample was ground again for further characterization.

According to Hai et al.^49^, the phosphor SMSO:Eu^2+^, Dy^3+^ was produced via a high-temperature solid-state process. The raw materials including Dy_2_O_3_ (99.999%), Eu_2_O_3_ (99.999%), SiO_2_ (A.R.), MgO (A.R.), SrCO_3_ (A.R.), and H_3_BO_3_ (A.R.) were weighed according to the stoichiometric proportions. After being thoroughly mixed for 20 min in agate mortars, the mixture was placed in alumina crucibles and heated at 1250 °C for 5 h under 5%H_2_/95%N_2_ environment. After being cooled to RT, the synthesized sample was ground again for further characterization.

### Characterization

XRD patterns were recorded using a Bruker D8 focus diffractometer. The morphology and elemental mapping were obtained by a scanning electron microscope (SEM, S-4800, Hitachi). The DR spectra were measured using a UV-vis-NIR spectrophotometer (Shimadzu, Japan). The PLE and PL spectra were obtained using a Hitachi F-7000 spectrophotometer. This spectrophotometer was also used to obtain the PersL spectra, the PersLE spectrum, and the PersL decay curves. Temperature-dependent PL spectra were collected on the Edinburgh Instruments FLS 920 spectrophotometer. IQEs were acquired using a Photonic Multi-channel Analyzer C10027 (Hamamatsu, Japan). The PersL digital photographs were recorded on a Canon EOS 600D. The homemade instrument composed of a heating device and a CCD detector was used to obtain TL spectra. XPS was carried out on a VG ESCALABMK II electron spectrometer. The EL performance was obtained using Starspec SSP6612 apparatus. The light flicker analyzer (Everfine Photo-E-Info Co. Ltd, LFA-3000) was used to assess the percent flicker of the AC-LEDs.

### AC-LED fabrication

Each AC-LED device was manufactured by combining a PiS made by dispersing the prepared phosphors in silicone with a 425 nm blue-emitting chip. The final AC-LED device was formed by curing the packaged device at 150 °C for 8 h.

## Supplementary information

SUPPLEMENTAL MATERIAL
