# Supplementary material for: Enhanced blue-light excited cyan-emitting persistent luminescence of BaLu2Al2Ga2SiO12:Ce3+, Bi3+ phosphors for AC-LEDs via defect modulation
Source: Light Sci Appl. 2022 Jun 17;11:184. doi: 10.1038/s41377-022-00868-8 (PMC9206004; doi:10.1038/s41377-022-00868-8)
Supplement: Supplementary file 1 — SUPPLEMENTAL MATERIAL [file 41377_2022_868_MOESM1_ESM.docx]

**SUPPLEMENTAL MATERIAL**

Enhanced blue-light excited cyan-emitting persistent luminescence of BaLu_2_Al_2_Ga_2_SiO_12_:Ce^3+^ phosphors for AC-LEDs via defect modulation

Weihong Yuan,^1,2^ Ran Pang,^1,*^ Shangwei Wang,^1^ Tao Tan,^1,2^ Chengyu Li,^1,^^2,3,*^ Chaowei Wang,^1^ and Hongjie Zhang^1,3^

*Correspondence: Ran Pang (pangran@ciac.ac.cn), Chengyu Li (cyli@ciac.ac.cn)*

*^1^ State Key Laboratory of Rare Earth Resource Utilization,* *Changchun Institute of Applied Chemistry, Chinese Academy of Sciences, Changchun 130022, China*

*^2^ University of Science and Technology of China, Hefei 230026, China*

*^3^ Zhongke Rare Earth (Guangzhou) Co., Ltd., Guangzhou 510700, China*

**Table S1** Refined structural data of BLAGSO, BLAGSO:0.05Ce^3+^, and BLAGSO:0.05Ce^3+^, 0.01Bi^3+^.

| Formula | BLAGSO | BLAGSO:0.05Ce^3+^ | BLAGSO:0.05Ce^3+^, 0.01Bi^3+^ |
| --- | --- | --- | --- |
| crystal system | cubic | cubic | cubic |
| space group | *Ia3d*(230) | *Ia3d*(230) | *Ia3d*(230) |
| *a* (Å) | 12.0483 | 12.0620 | 12.0648 |
| *b* (Å) | 12.0483 | 12.0620 | 12.0648 |
| *c* (Å) | 12.0483 | 12.0620 | 12.0648 |
| *α* = *β* = *γ* (deg) | 90 | 90 | 90 |
| *Z* | 8 | 8 | 8 |
| *V* (Å^3^) | 1748.952 | 1754.924 | 1756.137 |
| *R_p_* | 0.0450 | 0.0452 | 0.0460 |
| *R_wp_* | 0.0603 | 0.0606 | 0.0621 |
| *χ^2^* | 2.521 | 2.538 | 2.713 |

**Table S2** The atom positions of BLAGSO.

| atom | site | x | y | z | Occupancy | U_iso_ |
| --- | --- | --- | --- | --- | --- | --- |
|  |  |  |  |  |  |  |
| Ba1 | 24c | 0.125 | 0 | 0.25 | 0.3333 | 0.03211 |
| Lu1 | 24c | 0.125 | 0 | 0.25 | 0.6667 | 0.00257 |
| Al1 | 16a | 0 | 0 | 0 | 0.5790 | 0.00490 |
| Ga1 | 16a | 0 | 0 | 0 | 0.4210 | 0.03130 |
| Al2 | 24d | 0.375 | 0 | 0.25 | 0.2380 | 0.00100 |
| Ga2 | 24d | 0.375 | 0 | 0.25 | 0.4290 | 0.00100 |
| Si1 | 24d | 0.375 | 0 | 0.25 | 0.3330 | 0.00080 |
| O1 | 96h | -0.0399 | 0.049 | 0.1513 | 1.0000 | 0.01970 |

**Table S3** The atom positions of BLAGSO:0.05Ce^3+^.

| atom | site | x | y | z | Occupancy | U_iso_ |
| --- | --- | --- | --- | --- | --- | --- |
|  |  |  |  |  |  |  |
| Ba1 | 24c | 0.125 | 0 | 0.25 | 0.3333 | 0.01570 |
| Lu1 | 24c | 0.125 | 0 | 0.25 | 0.6506 | 0.01053 |
| Ce1 | 24c | 0.125 | 0 | 0.25 | 0.0161 | 0.80000 |
| Al1 | 16a | 0 | 0 | 0 | 0.5790 | 0.00990 |
| Ga1 | 16a | 0 | 0 | 0 | 0.4210 | 0.03630 |
| Al2 | 24d | 0.375 | 0 | 0.25 | 0.2380 | 0.00400 |
| Ga2 | 24d | 0.375 | 0 | 0.25 | 0.4290 | 0.00070 |
| Si1 | 24d | 0.375 | 0 | 0.25 | 0.3330 | 0.00590 |
| O1 | 96h | -0.038 | 0.0494 | 0.1517 | 1.0000 | 0.01910 |

**Table S4** The atom positions of BLAGSO:0.05Ce^3+^, 0.01Bi^3+^.

| atom | site | x | y | z | Occupancy | U_iso_ |
| --- | --- | --- | --- | --- | --- | --- |
|  |  |  |  |  |  |  |
| Ba1 | 24c | 0.125 | 0 | 0.25 | 0.3333 | 0.00940 |
| Lu1 | 24c | 0.125 | 0 | 0.25 | 0.6477 | 0.01035 |
| Bi1 | 24c | 0.125 | 0 | 0.25 | 0.0025 | 0.80000 |
| Ce1 | 24c | 0.125 | 0 | 0.25 | 0.0165 | 0.80000 |
| Al1 | 16a | 0 | 0 | 0 | 0.5790 | 0.00860 |
| Ga1 | 16a | 0 | 0 | 0 | 0.4210 | 0.03500 |
| Al2 | 24d | 0.375 | 0 | 0.25 | 0.2380 | 0.00400 |
| Ga2 | 24d | 0.375 | 0 | 0.25 | 0.4290 | 0.00070 |
| Si1 | 24d | 0.375 | 0 | 0.25 | 0.3330 | 0.00600 |
| O1 | 96h | -0.0377 | 0.0492 | 0.152 | 1.0000 | 0.02060 |


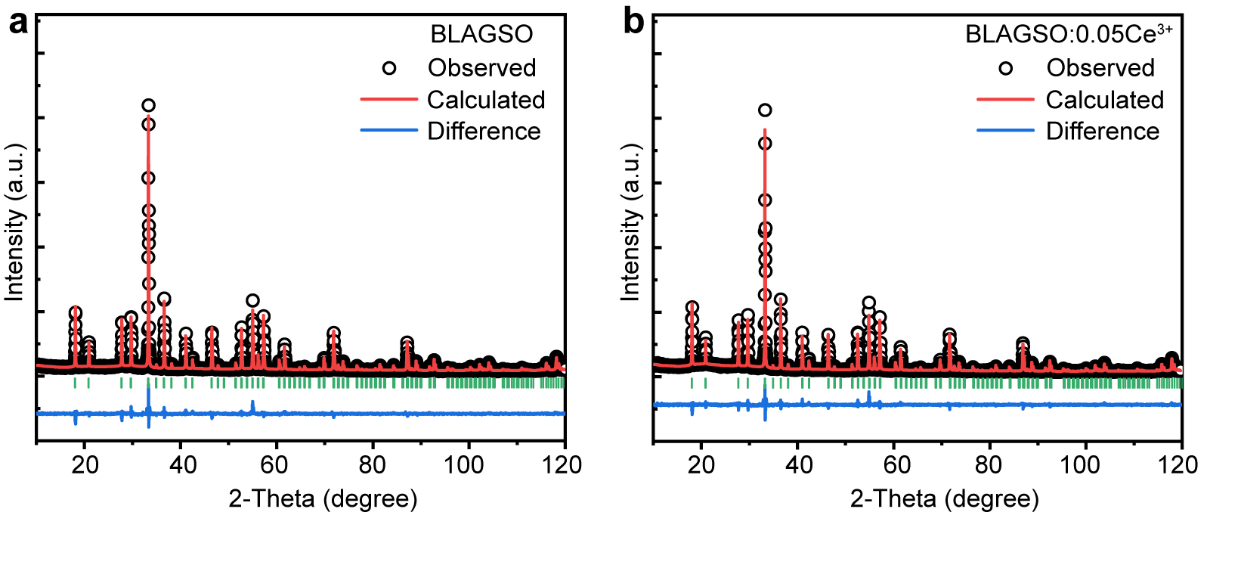


**Fig. S1** **a** – **b** XRD Rietveld refinement profiles for BLAGSO and BLAGSO:0.05Ce^3+^.


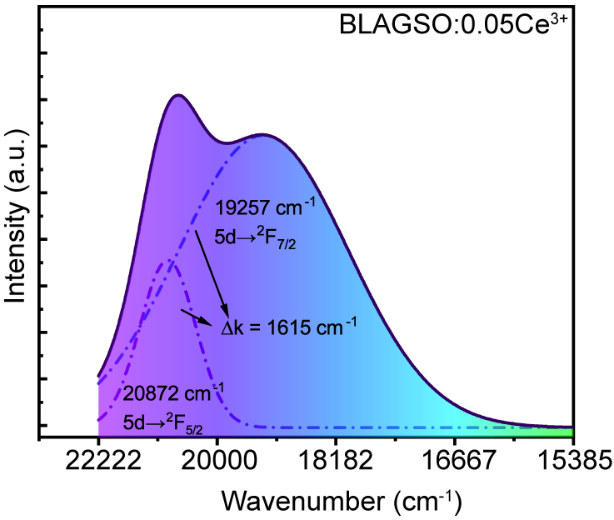


**Fig. S2** Deconvolution of emission spectra for BLAGSO:0.05Ce^3+^.


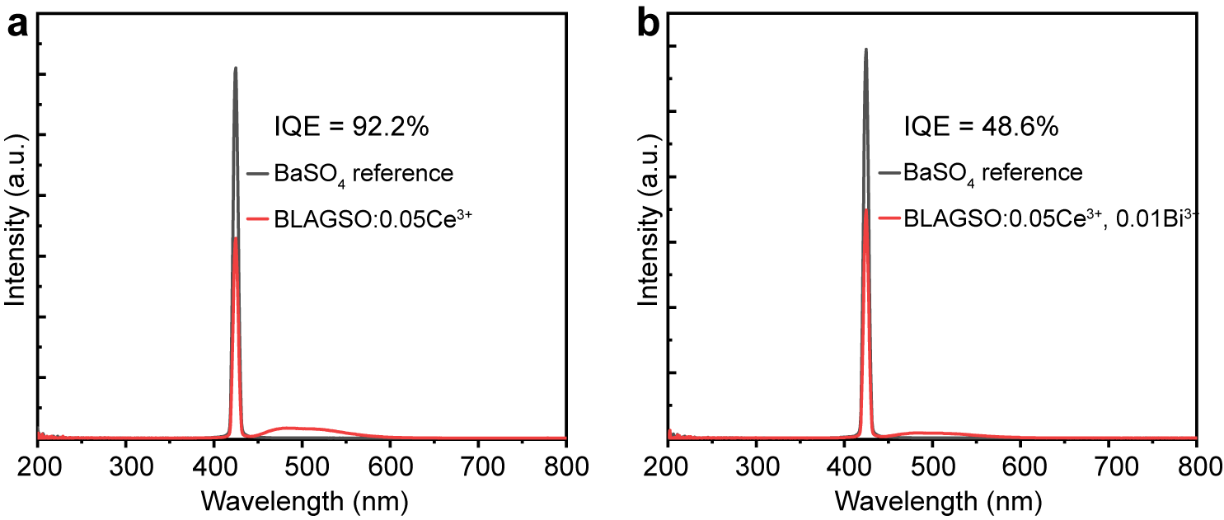


**Fig. S3** IQEs of BLAGSO:0.05Ce^3+^ and BLAGSO:0.05Ce^3+^, 0.01Bi^3+^.


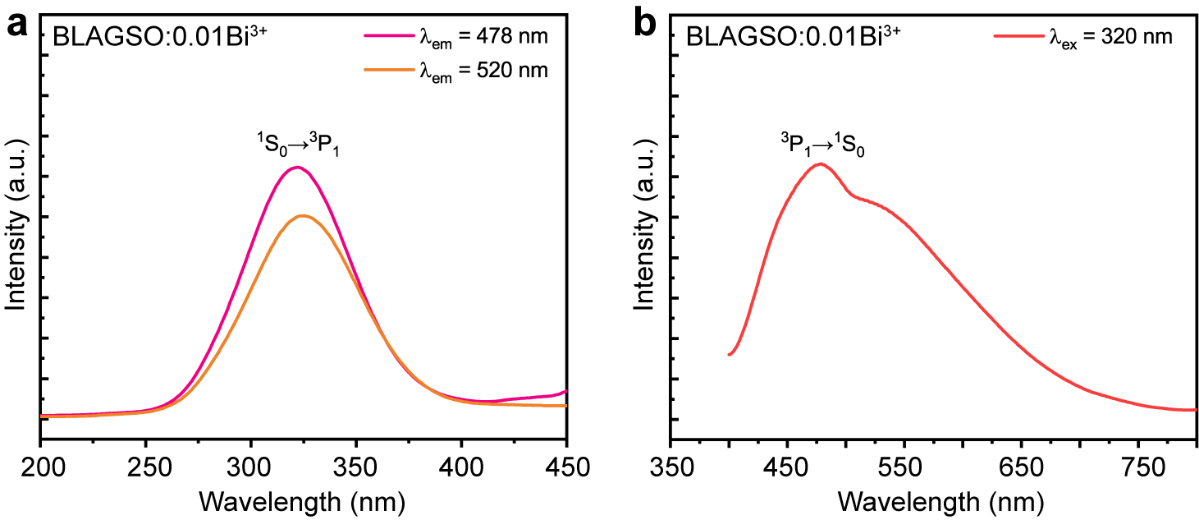


**Fig. S4 a** – **b** PLE and PL spectra of BLAGSO:0.01Bi^3+^.


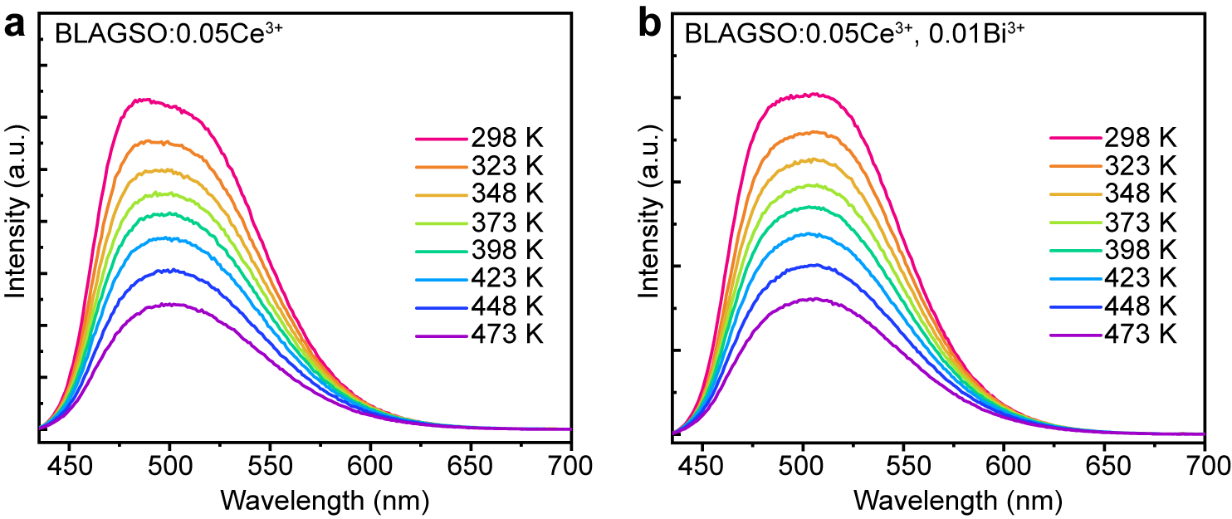


**Fig. S5 a** – **b** The corresponding temperature-dependent PL spectra.

**
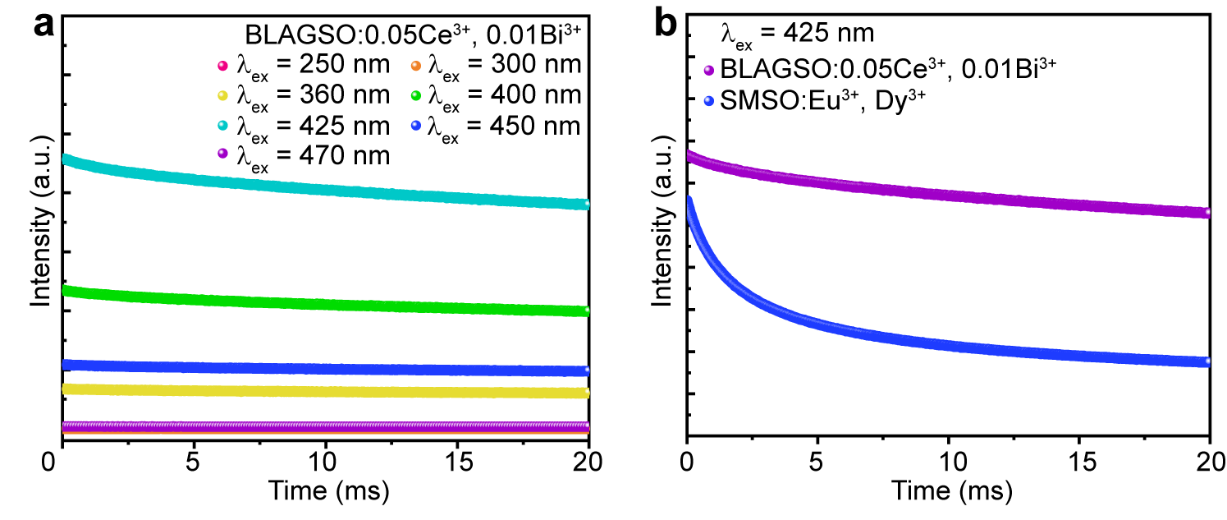
**

**Fig. S6 a** The representative PersL decay versus the excitation wavelength. **b** PersL decay curves of BLAGSO:0.05Ce^3+^, 0.01Bi^3+^ and SMSO:Eu^2+^, Dy^3+^.

**Supplementary Note**

Based on density functional theory (DFT), the band structure and electronic structure were calculated by Vienna ab initio simulation package (VASP) codes.^1,2^ The exchange correlation potential was approximated by generalized gradient approximation (GGA) using the Perdew–Burke–Ernzerhof (PBE) formulation.^3^ We used the projected augmented wave (PAW) potentials to describe the ionic cores.^4,5^ The plane wave basis set with a kinetic energy cutoff of 450 eV and we used 4×4×2 for the Monkhorst-Pack k-point for sampling the Brillouin zone during the whole computations. Partial occupancies of the Kohn−Sham orbitals were allowed using the Gaussian smearing method and a width of 0.05 eV. The electronic energy was considered to be self-consistent when the energy change was less than 10^-4^ eV. The geometric optimization was considered convergent when the force change was less than 0.05 eV/Å. Grimme’s DFT-D3 methodology was used to describe the dispersion interactions.^6^ The Hubbard parameter U was used to correct the exchange correlation energy for our systems. The f states in Ce, f states in Lu, and d states in Ba had been set as 4.51 eV, 4.26 eV, 3.91 eV.

**References**

1 Kresse, G. *et al.* Efficient iterative schemes for ab initio total-energy calculations using a plane-wave basis set. *Physical Review B* **54**, 11169-11186 (1996).

2 Perdew, J. P. *et al.* Generalized gradient approximation made simple. *Physical Review Letters* **77**, 3865-3868 (1996).

3 Kresse, G. *et al.* From ultrasoft pseudopotentials to the projector augmented-wave method. *Physical Review B* **59**, 1758-1775 (1999).

4 Blöchl, P. E. Projector augmented-wave method. *Physical Review B* **50**, 17953-17979 (1994).

5 Grimme, S. *et al.* A consistent and accurate ab initio parametrization of density functional dispersion correction (DFT-D) for the 94 elements H-Pu. *The Journal of Chemical Physics* **132**, 154104 (2010).

6 Henkelman, G. *et al.* A climbing image nudged elastic band method for finding saddle points and minimum energy paths. *The Journal of Chemical Physics* **113**, 9901 (2000).
